# Implementation of Non-Contact Temperature Distribution Monitoring Solutions for Railway Vehicles in a Sustainability Development System Transport

**DOI:** 10.3390/s22249624

**Published:** 2022-12-08

**Authors:** Arkadiusz Kampczyk, Wojciech Gamon, Katarzyna Gawlak

**Affiliations:** 1 Department of Engineering Surveying and Civil Engineering, Faculty of Geo-Data Science, Geodesy, and Environmental Engineering, AGH University of Science and Technology, al. A. Mickiewicza 30, 30-059 Krakow, Poland; 2 Department of Railway Transport, Faculty of Transport and Aviation Engineering, Silesian University of Technology, ul. Krasińskiego 8, 40-019 Katowice, Poland

**Keywords:** thermovision, thermal imaging, thermography, thermovision camera, temperature sensors, railway vehicle, railway rolling stock, railway infrastructure, railway suprastructure, non-traction needs, monitoring, surveying, diagnostics, measuring instruments

## Abstract

The implementation of temperature sensors represented by thermal imaging cameras is becoming increasingly rational. It is playing an important role in the socio-economic environment, in industry, scientific-research work. The main objective of the work is to assess the quality of the railway vehicles in exploitation and their thermal insulation, localise thermal bridges, and the tightness of the body using the FLIR-E6390 thermal inspection camera. An integration of test methods (research methods) was used including a diagnostic method based on a thermographic study integrated with the system approach method and system failure mode effects analysis (SFMEA). The scientific-research work included studies of seven types of railway vehicles in exploitation. A number of conclusions were reached. Specifically providing implementation of innovative and non-contact temperature distribution monitoring solutions for railway vehicles in a sustainability development system transport. Demonstrated the disparities between the different types of vehicles. Next, the identification of critical elements of their thermal insulation, the location of thermal bridges, and the tightness of the body of the rail vehicles, particularly the doors and inter-unit connections. The study covered the state of consumption of stationary electricity (for non-traction needs), implementing innovative indicators for stationary electricity consumption of railway vehicles as a new approach.

## 1. Introduction

The implementation of thermal imaging cameras is becoming increasingly rational. It is playing an important role in the socio-economic environment, especially in industry, scientific, and research work. With the rapid development of the rail transit industry, the safety and reliability of trains is a prerequisite to ensure the development of rail transit technology [1]. Fully functioning transport requires the integration of infrastructure and suprastructure. Railway infrastructure is the totality of railway facilities and equipment in their interconnection. Railway suprastructure comprises the means of transport, i.e., the railway rolling stock, including but not limited to: locomotives, coach passenger cars (passenger coaches), wagon freight cars (freight wagons), working vehicles, electric and diesel multiple units (trainset) and technical facilities. These guarantee the safe, regular and continuous movement of trains. Thus, Stypułkowski et al. in [2] state that the safety and reliability of railway transport require new solutions for monitoring and quick identification of faults in the railway infrastructure. Electric heating devices (EORs) are the crucial element of turnouts. Thermal imaging is a response to the need for an EOR inspection tool. Karakose et al. in [3] a complex fuzzy system-based thermography approach being proposed for predictive maintenance on electric railways. The experimental results obtained demonstrate the performance of the proposed complex fuzzy system and the performance achieved by processing thermal images. Dumoulin et al. in [4] referred to the outdoor thermal monitoring of large-scale structures by infrared thermography integrated into an iterative closest point (ICT) based architecture. An infrared system has been developed to monitor transport infrastructures in a standalone configuration. The autonomous configuration was also referred to by Dumoulin and Boucher in [5]. Dealing with infrared thermography system for transport infrastructures survey with inline local atmospheric parameter measurements and offline model for radiation attenuation evaluations. It should be emphasised that an infrared system has been developed to monitor transport infrastructures in a standalone configuration. It is based on low-cost infrared thermal cameras linked with a calculation unit in order to produce a corrected thermal map of the surveyed structure at a selected time step. A novel issue using a thermal imaging camera was presented by Berg et al. in [6]. Developed a method for detecting obstacles on the railway in front of a moving train using a monocular thermal camera. The proposed method includes a novel way of detecting the rails in the imagery, as well as a way to detect anomalies on the railway. Harmoniously an advanced thermal camera-based system for the detection of objects on rail tracks addressed is by Pavlović et al. in [7]. In contrast, Tan et al. in [8] presented a novel approach to evaluate the railway ballast fouling by using thermal imaging techniques. As well, Ramzan et al. in [9] dealt with pixel frequency-based railroad surface flaw detection using active infrared thermography for structural health monitoring (SHM). In this paper, Ghazanfari and Tehrani [10], numerical and experimental studies, including the finite element method, thermography, metallography, and hardness testing are performed to determine the characteristics of the welded UIC60 rail. Results, it is shown that four key parameters—such as the maximum temperature during the welding process, the total welding time, the upsetting time, and the upsetting force—control the size, microstructure, and the hardness profile of the heat-affected zone which directly affects the characteristics and quality of welding.

Thermal imaging, also known as thermography, is an investigation based on the measurement of the distribution of temperatures on the surface of an object or device under investigation. It is the most popular method of diagnosing objects and equipment, using the detection of infrared radiation. In thermal remote sensing, the invisible radiation patterns of objects are converted into visible images and these images are called thermograms or thermal images. Thermal images can be acquired using a portable, hand-held, or thermal sensors that are coupled with optical systems mounted on an airplane or satellite [11]. Ishimwe et al. in [11] highlight that this technology is a non-invasive, non-contact, and non-destructive technique used to determine thermal properties and features of any object of interest and therefore it can be used in many fields, where heat is generated or lost in space and time. It is widely used in the monitoring of suprastructure components as well as industrial machinery: processing (technological) as well as transport. This is confirmed by research conducted by Deilamsalehy et al. in [12], dealing with an automatic method for detecting sliding railway wheels and hot bearings using thermal imagery. In particular, the main contribution in [12] is the development of a computer vision method for automatically detecting the sliding wheels from images taken by wayside thermal cameras. The use of temperature sensors as well as thermal imaging cameras in the monitoring of rolling stock components were specifically addressed: Stoynova et al. [13] conducted a thematic electronic sensor system for monitoring the temperature status of rolling stock in motion, Sawczuk in [14] runs the evaluation of a rail disc brake braking process by using a thermal camera, Lisanti et al. in [15] presenting a multi-camera image processing and visualization system for train safety assessment. The system exploits high-rate visible and thermal cameras that observe a train passing under a railway overhead gantry. Next Kim et al. in [16] runs the study of the status monitoring system using the infrared camera for the key underbody components of railway vehicles (traction motors and reducers). In contrast, Deilamsalehy et al. in [17] have conducted research with sensor fusion of wayside visible and thermal imagery for rail car wheel and bearing damage detection. They used wayside thermal and visible spectrum cameras are one option for the automatic wheel and bearing inspection. They state this makes thermal and visible spectrum imagery complementary and if the images are fused the result will benefit from the strengths of both sensors. In turn, in [18] Deilamsalehy et al., they emphasise developing a computer vision method for automatically detecting the defects of rail car wheels using a wayside thermal camera.

The implementation of temperature sensors as well as thermal imaging cameras in the monitoring of industrial machine components were specifically addressed: Szurgacz et al. in [19] referred to the results of a study carried out to determine the thermal condition of a conveyor power unit using a thermal imaging camera. The measurements were taken with a thermal imaging diagnostic method which measures infrared radiation emitted by an object. They demonstrated that this technology provides a means of assessing the imminence and severity of a possible failure or damage. The method is a non-contact measuring technique and offers great advantages in an underground mine [19]. Glowacz in [20] took up fault diagnosis of electric impact drills using thermal imaging. An original method for feature extraction of thermal images BCAoID (Binarized Common Areas of Image Differences) is proposed. He stated that fault diagnosis based on thermal images can find applications for protecting rotating machinery and engines. Abdulshahed et al. in [21] presented thermal error modelling of machine tools based on an Adaptive Neuro-Fuzzy Inference System (ANFIS) with fuzzy c-means clustering using a thermal imaging camera. A new intelligent compensation system for reducing thermal errors of machine tools using data obtained from a thermal imaging camera is introduced. An Adaptive Neuro-Fuzzy Inference System with fuzzy c-means clustering (FCM-ANFIS) was employed to design the thermal prediction model. A very interesting research issue was presented by Carvalho et al. in [22] concerning a UAV-based framework for the semi-automated thermographic inspection of belt conveyors in the mining industry. They note that frequent and accurate inspections of industrial components and equipment are essential because failures can cause unscheduled downtimes, massive material, and financial losses or even endanger workers. In [22] enumerates and discusses the existing roller inspection techniques and presents a novel approach based on an unmanned aerial vehicle (UAV) integrated with a thermal imaging camera. Sztubecki et al. in [23] conclude that the current trends in monitoring areas, objects, and buildings aim to combine data obtained from various sources. Combining geodetic data with data obtained by other methods allows for the analysis of object changes in a broader aspect and increases the credibility of the engineering interpretation of the measurement results. At the same time Mrówczyńska et al. in [24] aptly state that regardless of the results and their interpretation, we should remember, the choice of the measurement method and methods of data processing is determined by the nature of the object and specific terrain conditions. In turn, Shvets in [25] notes that theoretical and experimental research are complementary and should be conducted together.

The scientific-research topic carried out could indicate at first glance that it is analogous to previous research carried out, due to the use of temperature sensors. However, closer and deeper research—including literature research—showed that the research topic undertaken in the implementation of non-contact temperature distribution monitoring solutions for railway vehicles in a sustainability development system transport had not previously addressed, while also representing a new innovative approach, implemented in civil engineering, surveying, and transport.

The main objective of the scientific-research work is to assess the quality of the railway vehicles in service (exploitation, operational) and their thermal insulation, as well as to localise thermal bridges (mostek termiczny, mostek cieplny in Polish) and the tightness of the railway vehicles body (bodywork, body) using temperature sensors, represented by the FLIR-E6390 thermal inspection camera. An integration of test methods (research methods) was used including a diagnostic method based on thermographic testing integrated with the system approach method and system failure mode effects analysis (SFMEA). The scientific-research work included studies of seven types of railway vehicles, actively in service, of production:

- latest (years 2017, 2018, and 2019);
- medium (years 2008, 2011, and 2012); and,
- older—senior (year 1975).

Accurate measurement of localised temperature changes is important for understanding thermal homeostatic phenomena and discovering future advanced diagnostics. The scientific-research work carried out has led to:

- assessing the quality of the rail vehicle body in exploitation and its thermal insulation, as well as the location of thermal bridges and the tightness of the railway vehicles body using temperature sensors, represented by the FLIR-E6390 thermal imaging camera;
- demonstration of sensitive body components of railway vehicles. The representative of which especially is the door area including the components around the mounting and the railway car interconnections (folding connections, inter-unit connections, interconnection);
- calculations average values for the temperatures of the body components of railway vehicles;
- demonstration of condition consumption of stationary electricity of railway vehicles (energy consumption for non-traction needs, including heating at standstill);
- definition and implementation of innovative indicators for stationary electricity consumption of railway vehicles (for non-traction needs) marked as *W_1_* and *W_2_* constituting a component of the new approach in the implementation of innovative and non-contact temperature distribution monitoring solutions for railway vehicles in a sustainability development system transport; and,
- to complement and fill an existing research gap that has not been addressed before, while at the same time providing a new and innovative approach, particularly for civil engineering, surveying, and transport.

The scientific-research work carried out has led to comprehensive conclusions confirming effectiveness, reproducibility, stability, fast response, feasibility, and accuracy. The excellent performance and simplicity of the implanted new approach are promising for civil engineering, surveying, and transport applications. The article was prepared under the research subvention of AGH University of Science and Technology No. 16.16.150.545 in 2022.

## 2. Related Work

A supplement to the ongoing theme of scientific-research work is the Related Work section. Making an essential contribution to the subject of implementation of innovative and non-contact temperature distribution monitoring solutions for railway vehicles in a sustainability development system transport. Constituting a presentation of the author’s contribution to scientific-research achievements in the context of related research areas. At the same time, Related Work contrasts, synthesizes, and provides a representation of the state of available knowledge in Temperature Sensors. Thus, in order to clearly understand the contribution of the scientific-research achievements included in the content of the publication, the presented areas of literature scientific-research remain correlated with them—presenting achievements—against the background of existing knowledge and providing an insight into their status. The areas of research included in Realted Work also represent the current limitations and need for implementation of innovative and non-contact temperature distribution monitoring solutions for railway vehicles in a sustainability development system transport. It is also very important to demonstrate the state of surrounding research and how it relates to the realised theme of the publication to the work of other disciplines and fields. The following paragraphs show the areas of research in relation to common monitoring themes in the:

- military, logistics, and industrial systems [26,27] and mobile multimedia devices, represented in particular by smartphones in non-contact smartphone-based monitoring of thermally stressed structures. Sefa et al. in [28] emphasise that the in-situ measurement of thermal stress in beams or continuously welded rails may prevent structural anomalies such as buckling. Advanced research applicable to specify components of communications construction is also referred to in the work of the [29,30,31]. The development of technology for the use of temperature sensors in integration with smartphones also confirms Hallock in [32] for dynamic infrared thermography and smartphone thermal imaging as an adjunct for preoperative, intraoperative, and postoperative perforator free flap monitoring. Subsequently, Hallock in [33] states that smartphone thermal imaging can enable the safer use of propeller flaps. Relatively recently, incredibly cheap but adequate thermal imaging cameras have become available that work in concert with the ubiquitous cell phone. In turn, Choi et al. in [34] presented thermal image enhancement using a convolutional neural network;
- in electrical power systems (EPS) and electronics [35,36,37]. At the same time, Żywiołek et al. in [38] state that energy conservation is a popular topic in scientific-research today, despite the specific effects of energy conservation awareness still being investigated. The results in [38] of the study clearly show that not only do economic factors affect energy saving, but also an informed society that cares about resource management;
- classification and monitoring of vehicles [39,40], road transport infrastructure [41,42], counting and detection of persons [43,44,45,46], and intelligent border security intrusion detection using the Internet of Things (IoT) and embedded systems [47];
- food supply and food quality [48], Rosak-Szyrocka and Abbase in [49] note and address the issues of how quality/safety managers can objectively and automatically implement the hazard analysis and critical control point (HACCP) principles of hazard analysis in the application of HACCP, which is the identification of risk priorities and of the related critical control points (CCPs). In turn, Ghosh et al. in [50] presented a quality evaluation of food by thermal imaging;
- medicine and in the aspect of the COVID-19 pandemic [51,52];
- volcanic processes and monitoring of urban green infrastructure [53,54].

Taking into account the scientific-research areas shown, it is concluded that our contribution to the subject matter of the work undertaken resolves existing limitations and has resulted in the implementation of innovative and non-contact temperature distribution monitoring solutions for railway vehicles in a sustainability development system transport.

## 3. Materials and Methods

Thermal insulation achieves thermal comfort and prevents energy loss, while guaranteeing reduced heat loss and effectively reducing noise levels. The identification of thermal bridges and airtightness of the railway vehicles, especially the railway vehicle body, makes it possible, on the basis of tests carried out, to locate components of the body whose thermal resistance is significantly reduced by the total or partial penetration of the railway vehicle body through materials with a different thermal conductivity coefficient by changing the thickness of the material layers.

**Figure 1:**
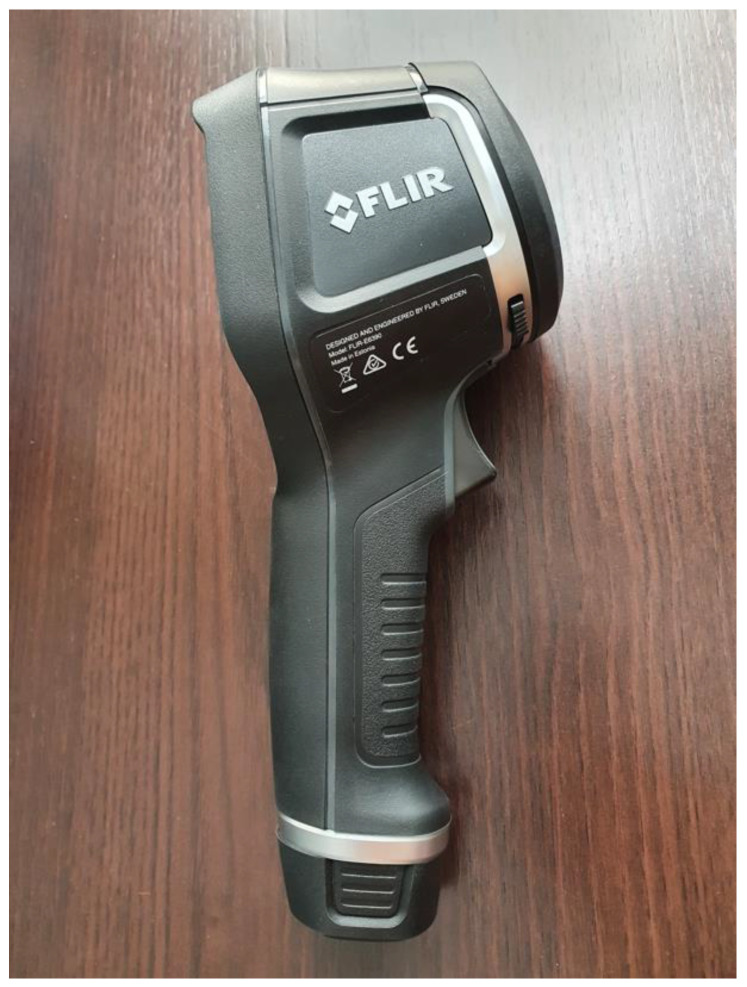
FLIR-E6390 thermal imaging camera used for scientific-research work.

The scientific-research work on the topic of implementation of non-contact temperature distribution monitoring solutions for railway vehicles in a sustainability development system transport was carried out using a diagnostic method based on thermographic surveys. With the application of temperature sensors, represented by the FLIR-E6390 thermal imaging camera (Figure 1), the characteristics of the technical parameters are shown in Table 1 [55]. The parameters of the FLIR-E6390 measuring device have been defined to ensure that studies can be carried out without having to change them during measurements. The emissivity value ε (base) in the initial stage was 0.98, the measured temperature range was −20.0–250.0 °C, and the measurement distance (base) in the initial stage was 1.0 m. However, it should be noted that during the analysis of the thermograms using the software provided by the manufacturer, the distances and emissivity values were adapted according to the areas studied, for example, a correction has been made to the measuring distance of 5.0 m for outdoor measurements.

The diagnostic method used, based on thermographic surveys, was integrated with the system approach method and system failure mode effects analysis. At the same time, referring to the whole and the interrelationships between the various components and the analysis of systems and subsystems remaining in correspondence between them.

The scientific-research work included in the implementation of innovative and non-contact temperature distribution monitoring solutions for railway vehicles in a sustainability development system transport was carried out on vehicles in continuous operation, categorised as passenger railway vehicles. At the time of the study, the vehicles were located on the standby tracks of the limited liability company Koleje Śląskie (Silesian Railways, Koleje Śląskie in Polish)—which is the main operator of passenger rail transport, particularly within the Silesian Voivodeship in Poland. The field research work was carried out during the winter period, i.e., from December 2020 to February 2021, including fieldwork using a diagnostic method based on thermographic surveys from the winter period—February 2021. The outdoor temperature at the test site was −8.0 °C (temperature monitoring took place using the temperature sensors contained in the FLIR-E6390 thermal imaging camera), under conditions of moderate cloud cover. No fog was observed during the scientific-research work. The relative humidity on the day was 80%, with no significant icing on the test surfaces. All the railway vehicles included in the scientific-research work were prepared by the passenger transport operator Silesian Railways to leave the railway siding to operate regular train services. The railway vehicles studied in the implementation of innovative and non-contact temperature distribution monitoring solutions for railway vehicles in a sustainability development transport system were put into operation and warmed up to receive passengers, with the quality and comfort of the journey ensured. In contrast, the temperature value inside the railway vehicles was in the range of 19.0–21.0 °C (temperature monitoring took place on the basis of the onboard internal temperature sensors of the individual railway vehicles).

The scientific-research work included studies of seven types of railway vehicles, actively in service, of production:

- latest (years 2017, 2018, and 2019);
- medium (years 2008, 2011, and 2012); and,
- older—senior (year 1975).

The selection of railway vehicles was chosen in such a way as to cross-check a wide range of vehicle types exploitation by passenger carriers, applicable to both domestic and international markets. The scientific-research work, therefore, covered vehicles in exploitation: the latest, medium, and older so-called senior vehicles—those in continuous use (Table 2).

Thermograms were taken from both the interior and exterior of the vehicles, subjected to further examination in FLIR Thermal Studio software. At the same time, the integration of the test methods used required an analysis of the technical documentation of the vehicle components tested, including the technologies and materials used by the manufacturers in, for example, the thermal insulation of the vehicles (Table 3).

## 4. Results

The integration of methods used in the work carried out the implementation of non-contact temperature distribution monitoring solutions for railway vehicles in a sustainability development system transport provided thermograms of critical components of the body structure, represented in particular by the vehicle doors and the folding joints connecting individual vehicle sections (inter-unit connections). These sites have been identified as the sources of the greatest heat loss reflected in the deterioration of in-service railway vehicles and their thermal insulation. Then the location of thermal bridges and railway vehicles body (railcar body) leakage. They originate both from the interconnection of individual vehicle components and the need for manufacturers to use insulating materials to a limited extent, due to the nature of these components and their exploitation work in the vehicle.

### 4.1. Sensitive Body Components of Railway Vehicles

**Figure 2:**
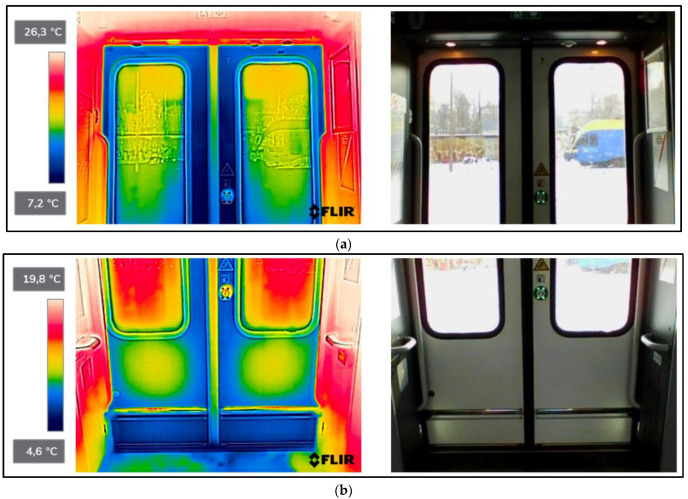

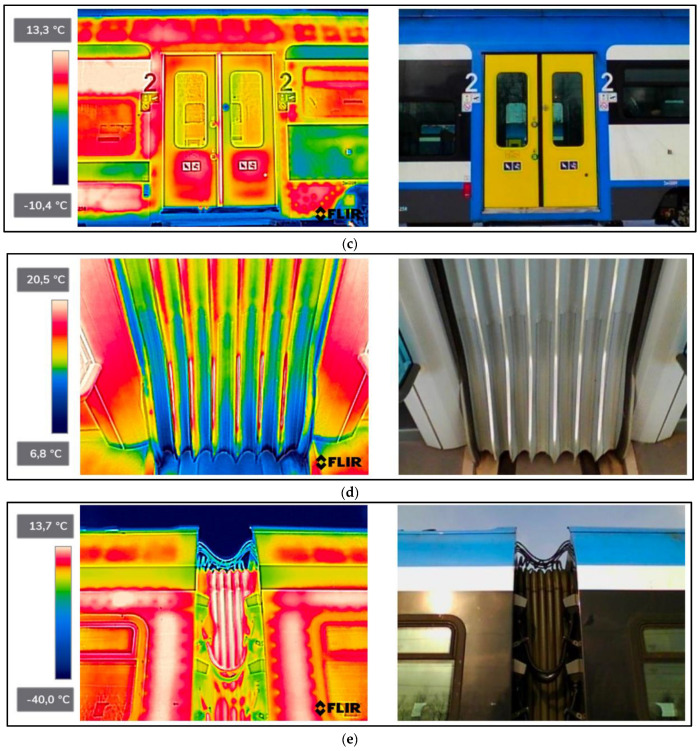
21WEa-003−thermogram of critical components of the railway vehicle: (**a**) door−upper part from inside the vehicle; (**b**) door−lower part from inside the vehicle; (**c**) door from outside the vehicle; (**d**) interconnection from inside the vehicle; (**e**) interconnection from outside the vehicle.

**Figure 3:**
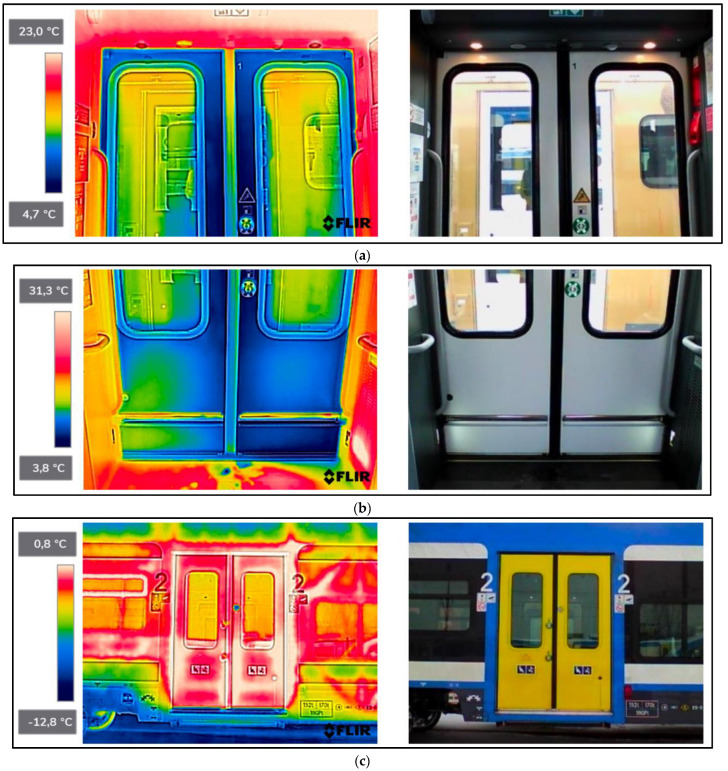

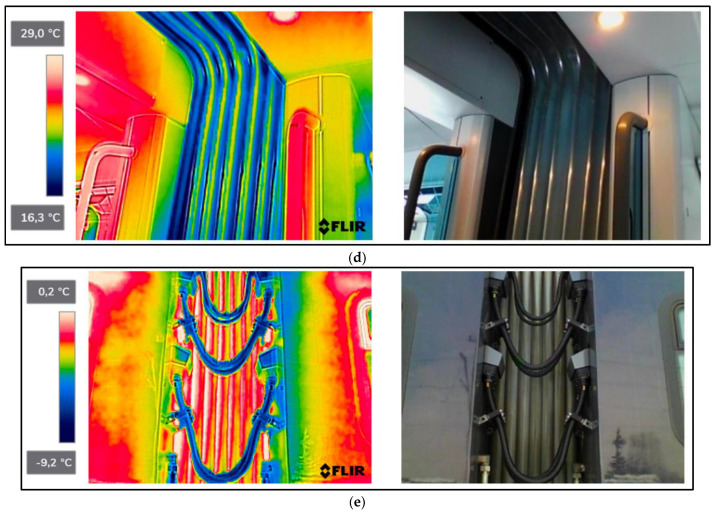
22WEd-011−thermogram of critical components of the railway vehicle: (**a**) door−upper part from inside the vehicle; (**b**) door−lower part from inside the vehicle; (**c**) door from outside the vehicle; (**d**) interconnection from inside the vehicle; (**e**) interconnection from outside the vehicle.

**Figure 4:**
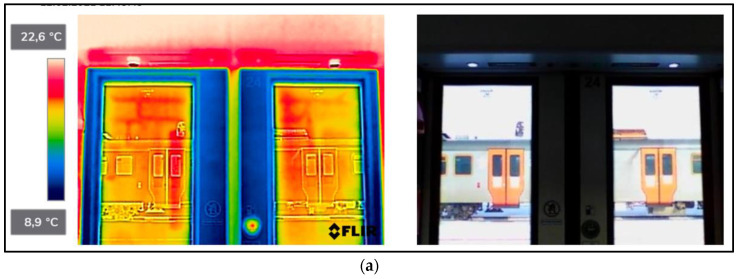

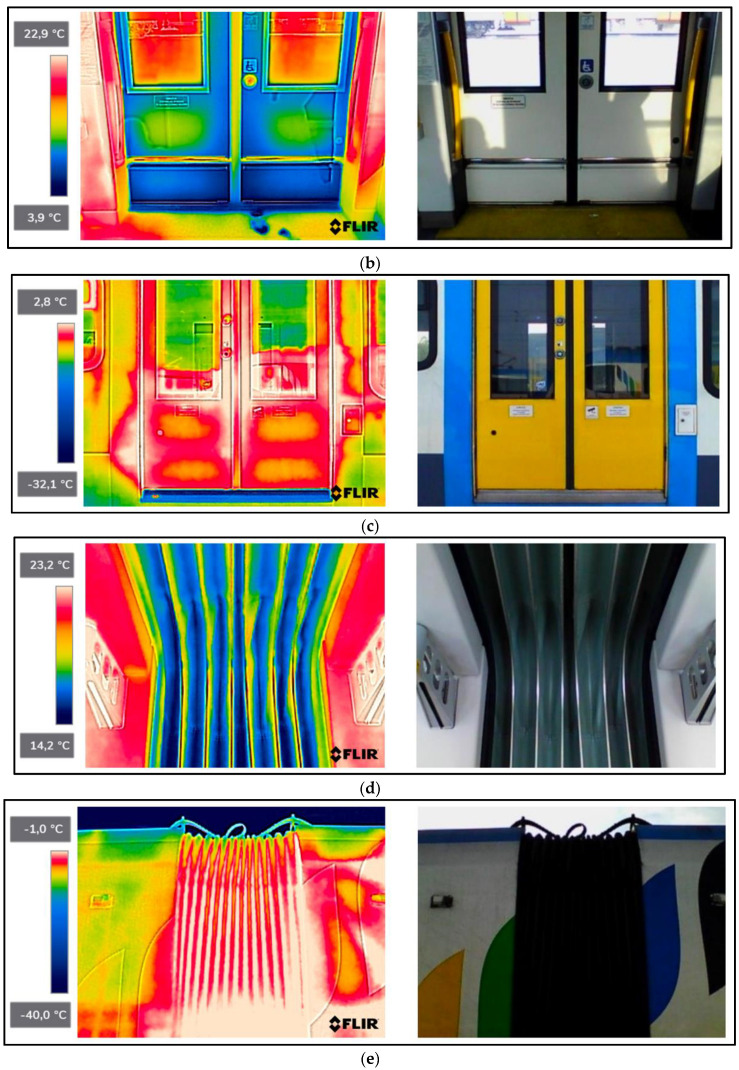
27WEb-003−thermogram of critical components of the railway vehicle: (**a**) door−upper part from inside the vehicle; (**b**) door−lower part from inside the vehicle; (**c**) door from outside the vehicle; (**d**) interconnection from inside the vehicle; (**e**) interconnection from outside the vehicle.

**Figure 5:**
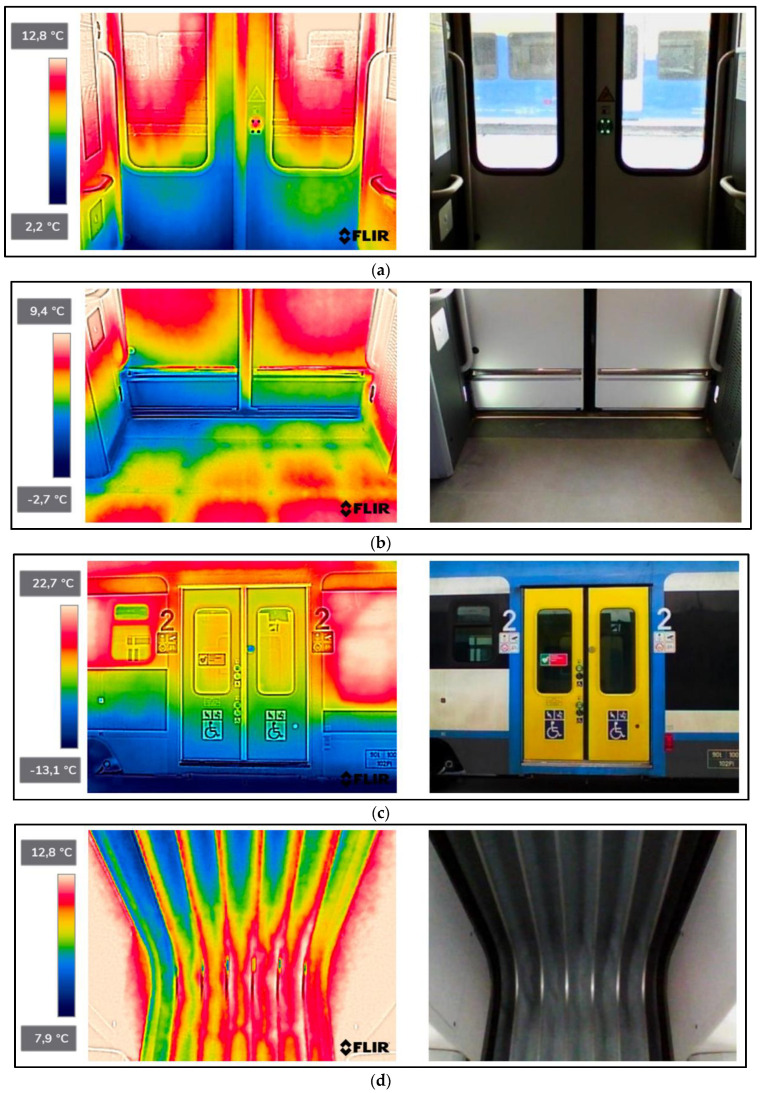

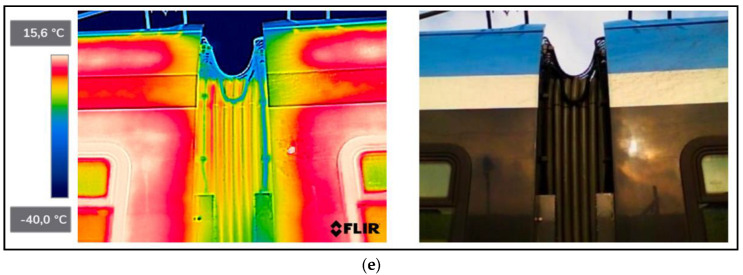
34WEa-004−thermogram of critical components of the railway vehicle: (**a**) door−upper part from inside the vehicle; (**b**) door−lower part from inside the vehicle; (**c**) door from outside the vehicle; (**d**) interconnection from inside the vehicle; (**e**) interconnection from outside the vehicle.

**Figure 6:**
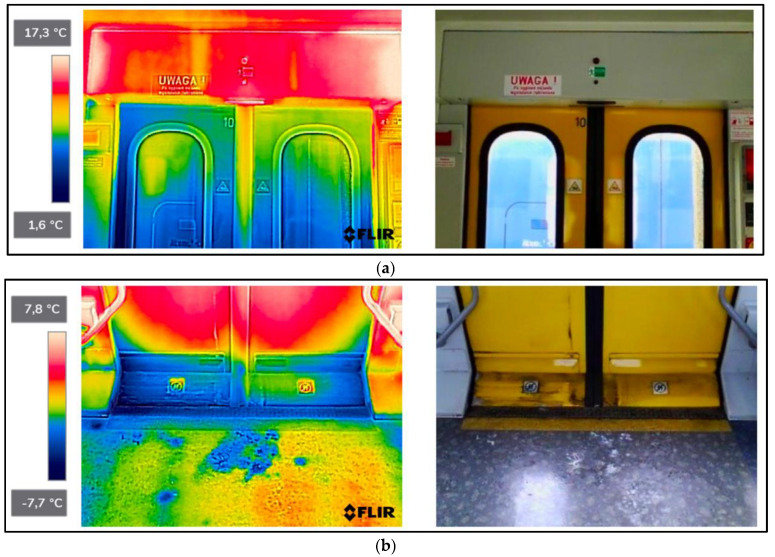

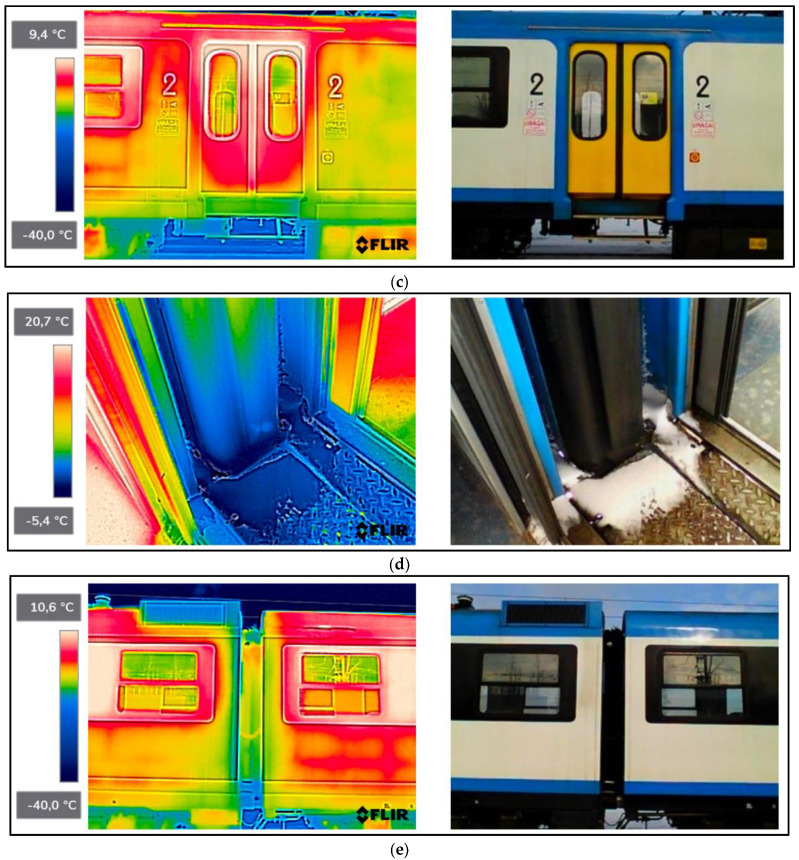
EN57AKŚ-223−thermogram of critical components of the railway vehicle: (**a**) door−upper part from inside the vehicle; (**b**) door−lower part from inside the vehicle; (**c**) door from outside the vehicle; (**d**) interconnection from inside the vehicle; (**e**) interconnection from outside the vehicle.

**Figure 7:**
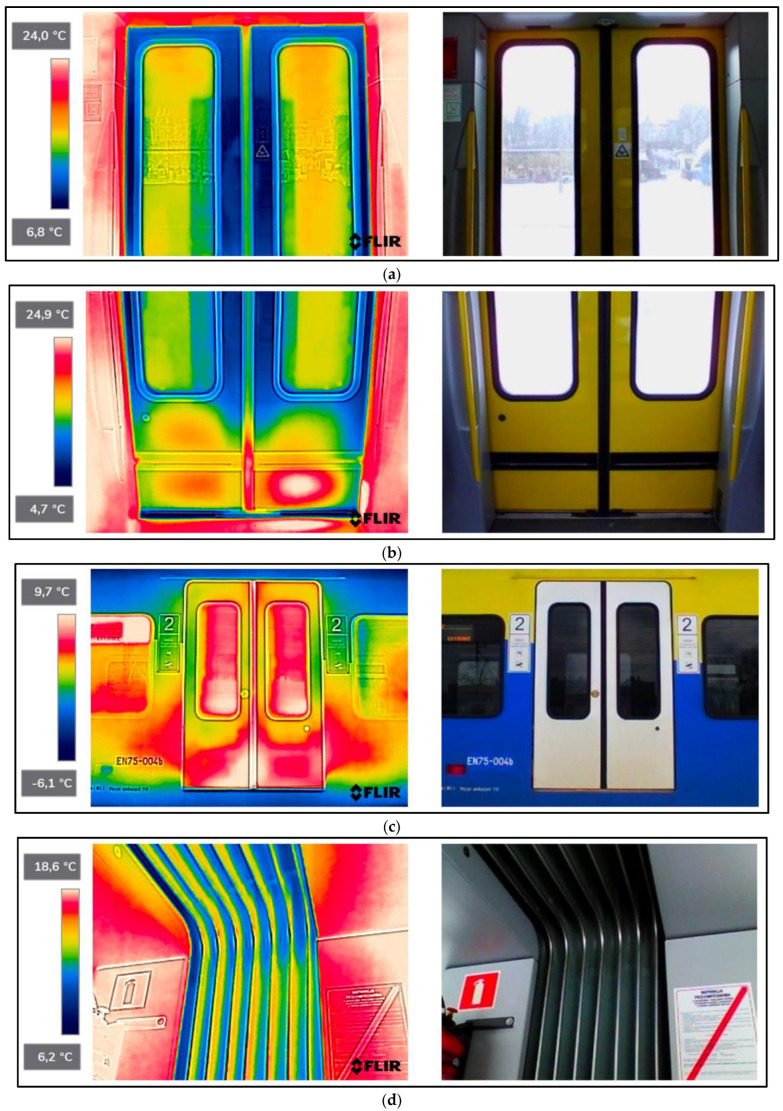

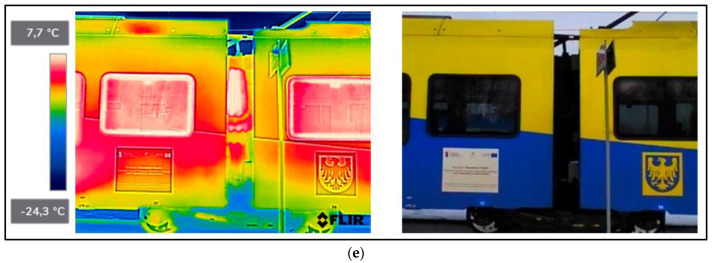
EN75-004−thermogram of critical components of the railway vehicle: (**a**) door−upper part from inside the vehicle; (**b**) door−lower part from inside the vehicle; (**c**) door from outside the vehicle; (**d**) interconnection from inside the vehicle; (**e**) interconnection from outside the vehicle.

**Figure 8:**
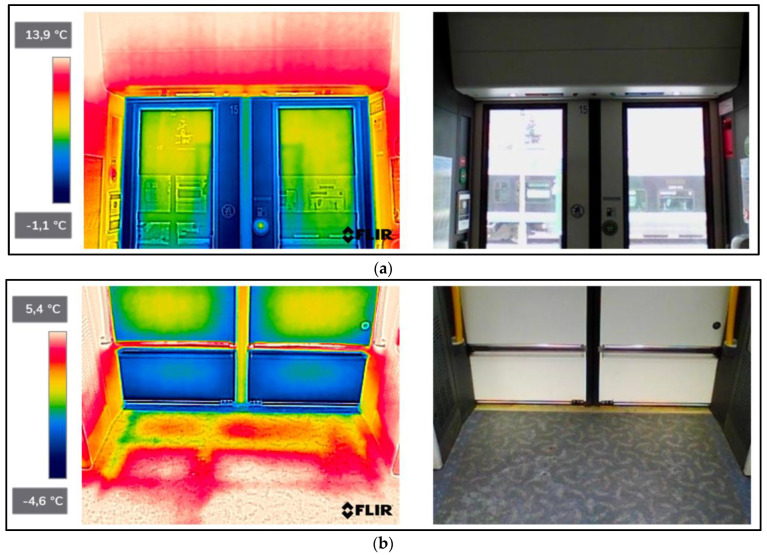

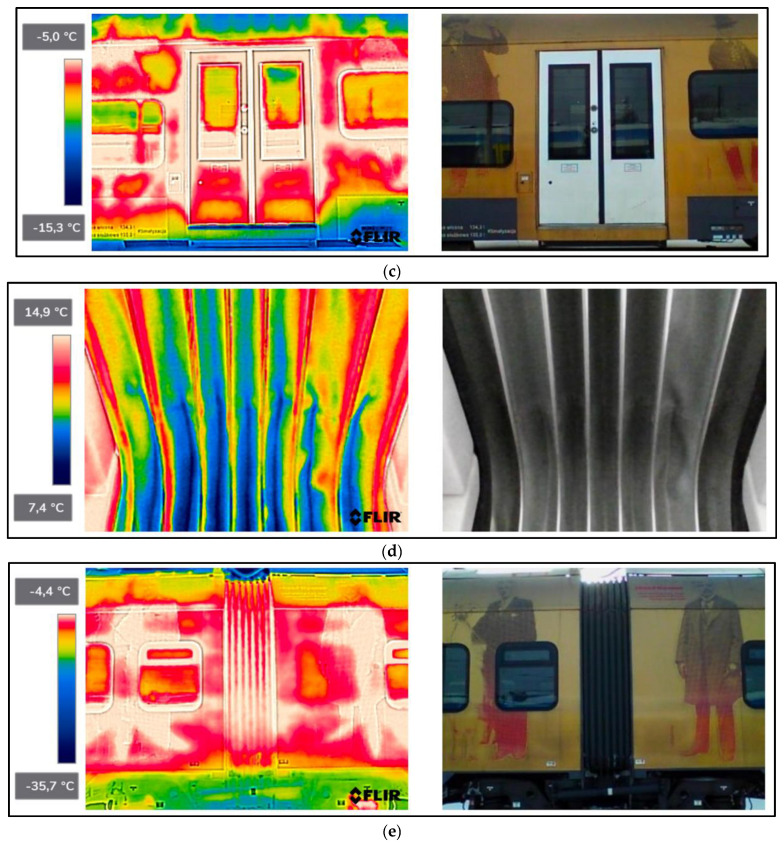
EN76-009−thermogram of critical components of the railway vehicle: (**a**) door−upper part from inside the vehicle; (**b**) door−lower part from inside the vehicle; (**c**) door from outside the vehicle; (**d**) interconnection from inside the vehicle; (**e**) interconnection from outside the vehicle.

As a result of the scientific-research work carried out, thermograms of seven railway vehicles were obtained (Table 2). The thermographic surveys included, for each vehicle, the doors to the passenger compartment and the inter-unit connections. Research on the doors, including the components around the assembly (montage), was carried out on the upper and lower parts from the inside of the vehicle and completely from the outside of the vehicle. Then the interconnections from inside and outside (Figure 2, Figure 3, Figure 4, Figure 5, Figure 6, Figure 7 and Figure 8). However, it should be stressed that, due to the different materials of the vehicles and thus their different emissivity values, the temperature values recorded at this stage have an instructive character. Due to the impossibility of using several emissivity values within one thermogram, Figure 2, Figure 3, Figure 4, Figure 5, Figure 6, Figure 7 and Figure 8 do not show the measured temperature values and the colour scale on the left side of the figure is for illustrative purposes. Figure 2, Figure 3, Figure 4, Figure 5, Figure 6, Figure 7 and Figure 8 show an approximation of the temperature distribution of the tested elements, which makes a graphic interpretation of the areas characterised by higher and lower values. The similar emissivity values of all the components tested (painted components, rubber components, veneered components) correlate with the illustrative nature of the thermograms shown. The exact values of the measured temperatures of the individual areas (for which actual emissivity values were used) are included in Table 4. For each thermogram, for greater clarity, the MSX Multi-Spectral Dynamic Imaging function was applied, which allows the merging of a visible light and thermal image.

The scientific-research work carried out on the basis of the thermograms in Figure 2, Figure 3, Figure 4, Figure 5, Figure 6, Figure 7 and Figure 8 showed the door area including the components around the montage and the inter-unit connections for all the studied vehicles to be critical places. Characterised by relatively low insulation properties in relation to the rest of the vehicle body. At the same time, these are the places where the greatest heat loss occurs. The study showed that, for all vehicles, a clear difference was discernible between areas of elevated temperature (thermograms taken from the outside of the vehicles) within the doors (under the windows) to areas located at the same height, but located in the area of the vehicle body where there is no front door (Figure 2c, Figure 3c, Figure 4c, Figure 5c, Figure 6c, Figure 7c and Figure 8c). These studies confirm that, in the case of vehicle doors, the lower part of the door has significantly worse insulating properties than the rest of the body (the exception to this rule is vehicle 34Wea-004, for which no such significant temperature difference between the lower part of the door and the rest of the vehicle body was recorded). This is due to the use of low-performance materials, also necessitated by the design of the door and its exploitation importance. Also having confirmation of the technical characteristics of the materials used in the individual railway vehicles (Table 3), including the lack of information in the technical documentation about the insulation materials used in the door construction. It should be emphasised that the work carried out showed clear thermal bridging in the area at the base of the vehicle entrance door. It is stated that this is due to the difficulty of sealing these elements, which, due to the way they are exploited, must be able to move undisturbed to allow the exchange of passenger streams (traffic flow). The specific consequences of thermal bridges are identified in the thermograms in the area of the floor, directly near the base of the vehicle doors. In addition, the research has shown here that there are significant differences between the various types of railway vehicles studied. The effects of this symptom are particularly pronounced in the thermograms for older vehicles of the older—senior type EN57AKŚ-223 (Figure 6). Due to the markedly different door design and operating principle, the thermal bridge formed at the base of the door has a significant effect on lowering the temperature of the vehicle interior, close to the floor. However, in the case of a vehicle EN75-004 (Figure 7) the best insulating properties of this area are found with respect to all the vehicles studied.

### 4.2. Average Values for the Temperatures of the Body Components of Railway Vehicles

**Figure 9:**
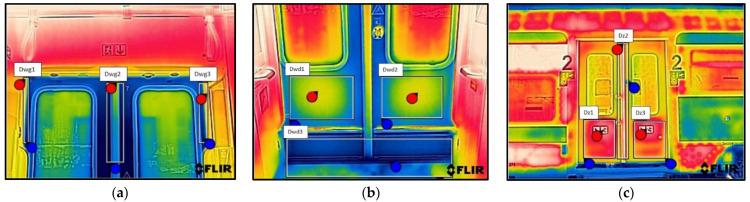
Defined locations for calculating the average door temperature value: (**a**) the upper part from inside the vehicle—Dwg1, Dwg2, Dwg3; (**b**) the lower part from inside the vehicle—Dwd1, Dwd2, Dwd3; (**c**) the upper and lower parts on the outside of the vehicle—Dz1, Dz2, Dz3. where: 🔴—the red dots indicate the locations with the highest recorded temperature; 🔵—the blue dots indicate the locations with the lowest recorded temperature.

**Figure 10:**
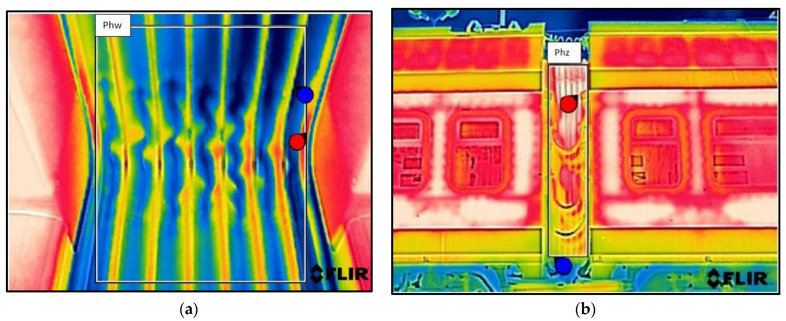
Defined locations for the calculation of the average temperature of the interconnections: (**a**) from inside the vehicle—Phw; (**b**) from outside the vehicle—Phz. where: 🔴—the red dots indicate the locations with the highest recorded temperature; 🔵—the blue dots indicate the locations with the lowest recorded temperature.

The obtained thermograms for individual types of railway vehicles were further processed in FLIR Thermal Studio software for further evaluation (Figure 2, Figure 3, Figure 4, Figure 5, Figure 6, Figure 7 and Figure 8). The average value of the temperatures of the individual areas of the thermograms for the key components of the railway vehicles was calculated. It should be emphasised that before analysing the temperature values in the software provided by the manufacturer, a detailed calibration of the parameters that affect the results was carried out. Exact emissivity values were used for the individual areas analysed (obtained from a reference measurement using a thermocouple—their value is indicated in Table 4), a correction has been made to the measuring distance (5 m for outdoor measurements) and accurate values for ambient and air temperature have been introduced (19 °C for indoor measurements and −8 °C for outdoor measurements), the temperature of camera optics (−5 °C—an average value has been taken due to the instantaneous movement of the thermal imaging camera to the inside and outside of the vehicles) and the relative humidity value (80%). The approach taken ensured that a comparative analysis of the thermal insulation properties of the components was carried out, thereby assessing them in terms of potential heat loss and energy loss. The locations defined for the calculation of the average temperature values for the individual tested surfaces of all railway vehicles are shown as an example of a vehicle 21WEa-003, respectively for: upper, and lower parts from inside the vehicle (Figure 9a,b), then for the door from outside the vehicle (Figure 9c). In the next stage, for inter-unit connections from inside the vehicle (Figure 10a) and from outside the vehicle (Figure 10b). For all thermograms of the tested vehicles, the places defined for calculating the mean temperature value were analogous. The areas of temperature measurement were chosen so that the surface to be tested had a uniform emissivity value.

**Figure 11:**
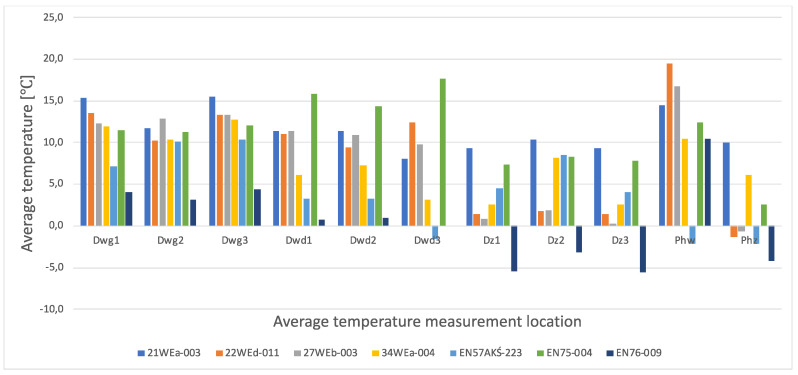
Graphical interpretation of the average component temperatures of the various types of railway vehicles, where: Dwg1.2.3−the places for measuring the inside of the door in the upper part; Dwd1.2.3−the places for measuring the inside of the door at the in the bottom par; Dz1.2.3—door measuring places from the outside; Phw−place of measurement of the interconnection from inside; Phz−the place of measuring the interconnection from outside.

The results of the calculation of the average temperature values of the individual areas of the thermograms for the key elements of the individual railway vehicle types are included in Table 4. In turn, their graphical interpretation is shown in Figure 11. In Table 4, the exact emissivity values of the individual areas for which the average temperature was measured are also included. Due to the use of similar materials (rubber, paint finish, veneers) in all the components analysed, a single, constant emissivity value was assumed for the individual areas, irrespective of the type of vehicle. In the case of the Phw area, an emissivity corresponding to the rubber elements was adopted, due to their far greater share of the thermogram area, compared to the metallic elements constituting the ribs. The thermogram in this area (Figure 2d, Figure 3d, Figure 4d, Figure 5d, Figure 6d, Figure 7d, Figure 8d and Figure 10a) shows a large variation in the emitted radiation due precisely to the heterogeneity of the materials used.

The average values of the temperatures of the individual areas of the thermograms for the key components of the different types of railway vehicles show significant differences in temperature for the individual components tested in the different types of railway vehicles. These differences are particularly noticeable in the case of vehicles of the type EN76-009 and EN57AKŚ-223.

#### 4.2.1. Average Values for Rail Vehicles Door Temperatures—From inside the Vehicle

When the temperature of the upper part of the doors is measured (thermograms taken from the interior of the vehicles), the average temperature for all types of vehicles (except for the two types shown above, i.e., EN76-009 and EN57AKŚ-223) is at a similar level and reaches values ranging from 10.0–16.0 °C. However, in the case of a vehicle, EN76-009 average temperatures are noticeably lower, ranging from 3.1–4.4 °C. In the case of a vehicle EN57AKŚ-223, the average temperature in this area is the second lowest of the vehicles tested, but is not significantly different from the overall group analysed. A lower value for the average temperature of the area in question is evident for measuring area ‘Dwg1’, demonstrating design and assembly errors and inaccurate adhesion of the door seal to the vehicle body on its left-hand side. In the case of the measurement of the average temperature of the lower part of the doors (thermograms taken from the interior of the vehicles), the results are analogous—there is a noticeable difference for vehicles EN76-009 and EN57AKŚ-223 compared to the rest of the group. Particularly evident for the ‘Dwd3’ measurement area (the lowest part of the door and the part of the vehicle floor adjacent to them) in the case of vehicle EN57AKŚ-223, where the recorded average temperature of the area is −1.6 °C. This demonstrates the unusual design of the door for this type of vehicle and its closing mechanism, resulting in the formation of a pronounced thermal bridge in the area of the floor, immediately adjacent to the vehicle door. In contrast, vehicle EN75-004 is found to have significantly higher values for the recorded average temperature, indicating better insulating properties for this type of vehicle within the doors, including in particular their seals.

#### 4.2.2. Average Values for Rail Vehicles Door Temperatures—From outside the Vehicle

In the case of thermograms of doors taken from the outside of vehicles, a greater discrepancy is found in the recorded values of the mean temperature. Having to do with the different insolation at the time the measurements were taken, despite them being carried out on the same day and in comparable time epochs. It should be stressed that the temperature of the area of ‘Dz2’ is clearly higher than that of ‘Dz1’ and ‘Dz3’ for all of the vehicles studied. This is particularly noticeable in the case of vehicles of the type 34WEa-004, EN76-009, and EN57AKŚ-223. The rationale here is to select the location of the average temperature measurement within the door seal connecting its individual wings (panels). In these vehicles, a thermal bridge forms at this point due to the clearly inferior insulating properties compared to the rest of the door area. In the case of vehicles 21WEa-003, 22Wed-011, 27WEb-003, and EN75-004 no such phenomenon has been registered, stating on the better design of the seal connecting the door panels.

#### 4.2.3. Average Temperatures of Inter-Unit Connections of Railway Vehicles—From inside the Vehicle

In the case of measurements of the mean temperature of the harmonic junction (thermograms taken from inside the vehicle—designation of the measuring area ‘Phw’), the recorded values are similar for almost all vehicles, with the exception of EN57AKŚ-223. In this case, the recorded average temperature is significantly lower than the rest of the vehicle group, with an average of −2.1 °C. This fact is due to the different design of this component for this type of vehicle (Figure 6d), which creates a gap between the body of the vehicle and the external environment to such an extent that snow may even fall into the vehicle interior (snowstorm). Due to this type of inter-unit connection design, a huge thermal bridge is created in this area for the EN57AKŚ-223 vehicle, resulting in significant heat loss from the heated vehicle interior.

#### 4.2.4. Average Temperatures of Inter-Unit Connections of Railway Vehicles—From outside the Vehicle

In the case of the measurement of the average temperature value of the inter-unit connections of railway vehicles—from outside the vehicle (designation of the measuring area ‘Phz’), the effect of sunlight, which influenced the results obtained, must also be taken into account. It corresponds especially in the case of a vehicle 21WEa-003 (average temperature 10.0 °C), for which, during the measurements, the insolation was greater than for the rest of the group of vehicles studied. The results confirmed that, for this area, the lowest average temperature was recorded for the vehicles EN76-009 and EN57AKŚ-223.

### 4.3. Consumption of Stationary Electricity—For Non-Traction Needs of Railway Vehicles

**Figure 12:**
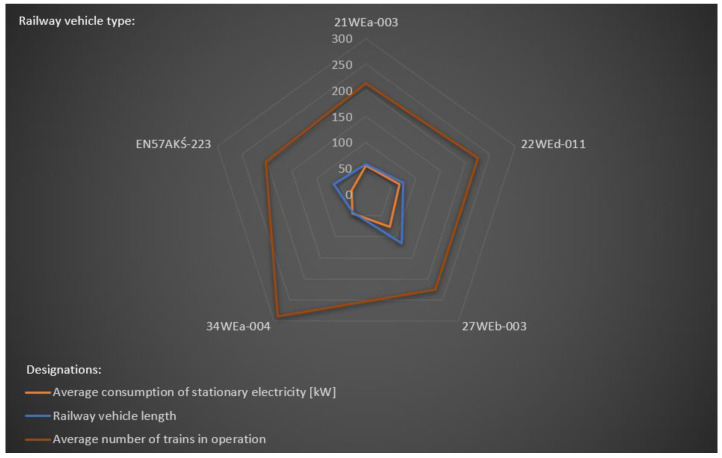
Consumption of stationary electricity of railway vehicles vs.: average consumption. of stationary electricity; railway vehicle length and average number of trains in operation.

The scientific-research work carried out in the implementation of innovative and non-contact temperature distribution monitoring solutions for railway vehicles in a sustainability development system transport included, in the final stage, the state of electricity consumption at standstill by the individual railway vehicles studied. The study covered a time horizon from December 2020 to February 2021, including fieldwork using a diagnostic method based on thermographic surveys from the winter period—February 2021. The average values of the amount of energy consumed by individual vehicles during stops were also related to the average number of trains running with these vehicles in a given period. Then to the length of the different types of vehicles *L_i_*. The new approach used is very significant and innovative, as it has the effect of demonstrating the state of energy consumption at standstill (greater volume of vehicle required to heat). The results of the study carried out on the standstill electricity consumption of the rail vehicles included in the study are presented in Table 5. A graphical interpretation of the consumption of stationary electricity against: the average consumption of stationary electricity, railway vehicle length *L_i_* and an average number of trains in operation is interpreted in Figure 12. It presents the energy consumption status of the five railway vehicles against which the study covered the full monitoring period. Two railway vehicles i.e., EN75-004 and EN76-009 were excluded from the interpretation due to a lack of complete data. In terms of the five interpreted vehicles in Figure 12, a trend is noticeable in 27WEb-003, of which the average consumption of stationary electricity is 76.33 [kW], at the same time in relation to railway vehicle length *L_27WEb-003_* = 114.75 m, at an average number of trains in operation amounting to 225. A competitive alternative is demonstrated by the vehicle 34WEa-004, of which the average consumption of stationary electricity is 44.67 [kW], at the same time in relation to railway vehicle length *L_34WEa-004_* = 42.83 m, at an average number of trains in operation amounting to 289. However, this is subject to the length of the train.

The results of the tests in Table 5 show that the average electricity consumption at stops in each winter month varies according to the type of vehicle. This is due to the different number of trains carried by each type of vehicle and their different length and volume, which have a significant impact on the energy consumption necessary to ensure a comfortable temperature for passengers (Figure 12).

Therefore, as a new approach, indicators were defined and implemented: *W_1_* and *W_2_*, which harmonise the recorded values for specific vehicle types.

### 4.4. Innovative Indicators for Stationary Electricity Consumption—For Non-Traction Needs of Railway Vehicles

The defined and implemented indicator *W_1_* indicates the average energy consumption per average number of trains running in the period under analysis (1):

(1) $$W_{1}=\frac{Average\;consumption\;of\;stationary\;electricity}{Average\;number\;of\;trains\;in\;operation}$$

At the same time, this indicator is dependent on the differences occurring when successive trains are operated by the types of vehicles concerned. Due to the work schedule resulting from the train timetable, there are transitions between trains of only a few minutes, in other cases, it can take up to several hours. Unfortunately, it is not possible to make more accurate calculations in this respect without an enormous amount of work (manual counting of individual stop times involving thousands of pieces of data), but this indicator nevertheless gives some information on a more accurate value of the actual energy consumption during a stop (Table 5).

In order to increase the state of illustration of the energy consumption data, the *W_1_* indicator was related to the length of the individual *L_i_* vehicles, illustrating the state of consumption (approximately) per volume that has to be heated for the individual vehicles (Table 3).

Finally, the definition of indicator *W_1_* ensured the implementation of indicator *W*_2_, shown by Equation (2):

(2) $$W_{2}=\frac{W_{1}}{L_{i}}$$

The innovative indicator indicated in Equations (1) and (2) used as a new approach in the implementation of innovative and non-contact temperature distribution monitoring solutions for railway vehicles in a sustainability development transport system provided the calculations contained in Table 3. From this it can be seen that the most favourable *W*_2_ indicator is achieved by vehicle type EN57AKŚ-223. The value of this result corresponds to the fact that in this type of vehicle in terms of measurement of electricity consumption is recorded by a meter characterised by an older design, compared to the other vehicles studied. In addition, it should be noted that this vehicle, due to its older design, lacks a number of features and equipment that newer type vehicles, powered at standstill, have. However, despite the lower value of the designated indicator, it should not be directly compared to the other vehicles in the group of vehicles analysed. Vehicles 21WEa, 22Wed, and 34WEa belonging to the same family (trade name ‘ELF 2’) have similar wear and tear (similar *W*_2_ index value). At the same time, it should be stressed that the vehicle type 27WEb-003, despite its significantly longer length compared to the other vehicles, has a lower index value than the aforementioned.

## 5. Discussion

The completed scientific-research topic in the implementation of non-contact temperature distribution monitoring solutions for railway vehicles in a sustainability development system transport confirms, that to date it has been characterised by a research gap that has not been addressed before. Its implementation constitutes representing a new and innovative approach, particularly for civil engineering, surveying, and transport—ensuring that the current research gap is filled. At the same time, it enriches the knowledge of target users, industry, and decision-makers from many countries, and professional circles, having a reflection on the quality and comfort of travel and the socio-economic environment.

One must rightly agree with the statement that the current trends in monitoring areas, objects, etc., aim to combine data obtained from various sources. Combining data with data obtained by other methods allows for the analysis of object changes in a broader aspect and increases the credibility of the engineering interpretation of the measurement results [23]. At the same time, the integration of scientific-research methods, then combining scientific and practical-expert knowledge, increases the quality of the research conducted. This is also confirmed by Shvets in [25] notes that theoretical and experimental research are complementary and should be conducted together. Thus, efficient implementation of the production process is important [56].

The scientific-research work carried out confirms the significant differences between the various types of railway vehicles. This is due to the use of different insulation materials and the different design of the vehicle itself. Against this backdrop, the least favourable results were recorded for the older type of vehicle, the EN57AKŚ-223, for which significant thermal bridges were identified, arising in particular around the base of the vehicle entrance doors. On the other hand, vehicles of types 21WEa, 22Wed, and 34WEa (belonging to the same family) appear to have an improved design (especially the door seals) compared to their predecessors—EN76 and 27WEb being an earlier family of vehicles produced by the same manufacturer. The EN76-009 vehicle is characterised by positive insulating properties of the components studied and, on the basis of the results obtained, it can be concluded that in this respect it has more favourable parameters than the other vehicles studied. The new indicators defined and implemented for average electricity consumption at standstill for non-traction needs do not coincide with the data obtained from the analysis of the thermograms. They show the most favourable indicator for the EN57AKŚ-223 vehicle. In the juxtaposition of the temperature surveys carried out and the thermal bridges identified, it appears to be contradictory. However, it is important to be aware that in new type of railway vehicles a significant proportion of the electricity is used not only for heating, but also to power other components and systems of the vehicle with which it is equipped. A similar debatable conclusion can be drawn from the energy consumption of the vehicles of the ‘ELF2’ and ‘ELF1’ families (EN76 and 27WEb). Older vehicles have fewer components requiring power at standstill, while translating into direct energy consumption values.

The applied integration of testing methods in combination with a diagnostic method based on thermal imaging technology in a sustainable transport system provides an excellent solution for the application of non-contact solutions for monitoring the temperature distribution of railway vehicle components. Specifically using infrared detection. Technology is a non-invasive, non-contact, and non-destructive technique used to determine the thermal properties and features of any object of interest and therefore it can be used in many fields, where heat is generated or lost in space and time [11]. An important aspect is that the implementation of innovative and non-contact temperature distribution monitoring solutions for railway vehicles in a sustainable transport system is significantly reflected in the understanding and protection of the state of energy consumption in correspondence with environmental protection. This aspect has not been more extensively analysed to date, which, in the context of the sustainable development of transport and its suprastructure, may prove to be a contribution to discussion and research in this area, making rail transport even closer to achieving climate neutrality.

## 6. Conclusions

The completed scientific-research work forms the basis for further research into the application of thermal imaging in civil engineering, surveying, and transport, particularly into the thermal insulation properties of railway vehicles, other suprastructure components, and infrastructure. At the same time, it fills a gap in the hitherto existing research field of the international gremium of scientific-research work, providing the beginning of a scientific debate on the topic of ongoing research. In summary, the scientific-research work carried out has led especially to:

- assessing the quality of the rail vehicle body in exploitation and its thermal insulation, as well as the location of thermal bridges and the tightness of the railway vehicles body using temperature sensors, represented by the FLIR-E6390 thermal imaging camera;
- demonstration of sensitive body components of railway vehicles. The representative of which especially is the door area including the components around the mounting and the railway car interconnections;
- identification of significant differences in thermal insulation between types of railway vehicles;
- calculations average values for the temperatures of the body components of railway vehicles;
- demonstrate that the older type of rail vehicle (EN57AKŚ-223) has significantly worse thermal insulation parameters than the other vehicles studied;
- to identify thermal bridges arising in individual vehicles (e.g., door seals, the base of interconnection);
- demonstration of condition consumption of stationary electricity of railway vehicles; and,
- definition and implementation of innovative indicators for stationary electricity consumption of railway vehicles marked as *W_1_* and *W_2_*.

As part of further scientific-research work, it is planned to extend the study to other types of railway vehicle exploitation by railway operators in the international market. This will be followed by the implementation of tests during the summer, during which the internal temperature is regulated using air conditioning.

## Figures and Tables

**Table 1:** Technical specifications of the FLIR-E6390 thermal imaging camera.

Parameter	Value
IR resolution	240 × 180 pixels
Thermal sensitivity/NETD	<0.06 °C (0.11 °F)/<60 mK
Field of view (FOV)	45° × 34°
Spatial resolution (IFOV)	3.4 mrad
Image frequency	9 Hz
Detector type	Focal plane array (FPA), uncooled microbolometer
Spectral range	7.5–13 μm
Operating temperaturę range	−15 °C to +50°
Accuracy (for ambient temperature 10 to 35 °C, object temperature above 0 °C and camera temperature range −20 to 250 °C	±2 °C

**Table 2:** List of railway vehicles subjected to studies.

Railway Vehicle Type	Factory Number	Year of Production	Producent
21WEa-003	E737BNA003 a, c, b	2017	PESA Bydgoszcz joint-stock company (PESA Bydgoszcz S.A. in Polish)
22WEd-011	E747BNA011 a, b, c, d	2019	PESA Bydgoszcz joint-stock company (PESA Bydgoszcz S.A. in Polish)
27WEb-003	E763BNA003 a, b, c, d, e, f	2012	PESA Bydgoszcz joint-stock company (PESA Bydgoszcz S.A. in Polish)
34WEa-004	E722BNA004 a, b	2018	PESA Bydgoszcz joint-stock company (PESA Bydgoszcz S.A. in Polish)
EN57AKŚ-223	311/001 a, s, b	1975	State-owned Pafawag Wagon Factory, NEWAG S.A. modernisation and Electric Locomotive Repair Plant in Gliwice (Państwowa Fabryka Wagonów Pafawag, modernizacja NEWAG S.A. oraz Zakłady Naprawcze Lokomotyw Elektrycznych w Gliwicach in Polish)
EN75-004	L-415811 a, b, c, d	2008	Stadler Poland limited liability company (Stadler Polska sp. z o.o. in Polish)
EN76-009	E742BNA009 a, b, c, d	2011	PESA Bydgoszcz joint-stock company (PESA Bydgoszcz S.A. in Polish)

**Table 3:** Technical characteristics of materials used in railway vehicles.

Railway Vehicle Type	Type of Material	
	Thermal Insulation	Interconnection
21WEa-003	Material: Plastotect (thickness 20 mm—floor ramps, 25 mm—lower deck, 50 mm—walls below window line), Moniflex (thickness 10 mm), and mineral wool (thickness 40 mm—overhead deck)	Folding sheet (harmonica) exterior/interior—fabric SEF3, light alloy profiles; rubber protective collar—EPDM
22WEd-011	Material: Plastotect (thickness 20 mm—floor ramps, 25 mm—lower deck, 50 mm—walls below window line), Moniflex (thickness 10 mm), and mineral wool (thickness 40 mm—overhead deck)	Folding sheet (harmonica) exterior/interior—fabric SEF3, light alloy profiles; rubber protective collar—EPDM
27WEb-003	Material: Moniflex—60 mm overhead deck, 60 mm sidewalls, 60 mm flooring	External/internal corrugated harmonica—harmonica fabric EF 5, M 3, T 5, and light alloy metal sections
34WEa-004	Material: Plastotect (thickness 20 mm—floor ramps, 25 mm—lower deck, 50 mm—walls below window line), Moniflex (thickness 10 mm), and mineral wool (thickness 40 mm—overhead deck).	Folding sheet (harmonica) exterior/interior—fabric SEF3, light alloy profiles; rubber protective collar—EPDM
EN57AKŚ-223	In the side walls and ceiling, mineral wool matting covered on one side with foil aluminium foil	No data available
EN75-004	No data available	Double harmonic for good thermal and acoustic insulation
EN76-009	Material: Moniflex—60 mm overhead deck, 60 mm sidewalls, 60 mm flooring	Outer/inner wavy harmonica—harmonica fabric EF 5, M 3, T 5, and light alloy metal sections

where: SEF3—Rubberised textile fabric, HÜBNER bellows fabric. EPDM—Ethylene Propylene Diene Monomer.

**Table 4:** Average component temperatures of the different types of railway vehicles.

Place of Defined Measurement		Railway Vehicle Type						
	Emissivity ε	21WEa-003	22WEd-011	27WEb-003	34WEa-004	EN57AKŚ-223	EN75-004	EN76-009
Average temperature value [°C]								
Interior door (upper part)								
Dwg1	0.96	15.4	13.5	12.3	11.9	7.1	11.5	4.1
Dwg2	0.93	11.7	10.2	12.9	10.3	10.1	11.3	3.1
Dwg3	0.96	15.5	13.3	13.3	12.7	10.3	12.1	4.4
Interior door (lower part)								
Dwd1	0.96	11.4	11.0	11.4	6.1	3.2	15.8	0.7
Dwd2	0.96	11.4	9.4	10.9	7.3	3.3	14.3	0.9
Dwd3	0.95	8.0	12.4	9.8	3.1	−1.6	17,7	0,0
Doors from outside								
Dz1	0.96	9.3	1.4	0.8	2.5	4.5	7.4	−5.5
Dz2	0.93	10.3	1.8	1.9	8.2	8.5	8.3	−3.2
Dz3	0.96	9.3	1.4	0.3	2.6	4.1	7.8	−5.6
Interconnection from within								
Phw	0.91	14.5	19.5	16.7	10.5	−2.1	12.4	10.4
Interconnection from the outside								
Phz	0.91	10.0	−1.3	−0.6	6.1	−2.1	2.5	−4.2

**Table 5:** Consumption of stationary electricity—For non-traction needs of railway vehicles—time horizon December 2020–February 2021.

Railway Vehicle Type	Average Consumption of Stationary Electricity [kW]	Average Number of Trains in Operation	Railway Vehicle Length *L_i_* [m]	Index Average Energy Consumption per Number of Trains in Operation *W_1_*	Index $W_{2}=\frac{W_{1}}{L_{i}}$
21WEa-003	55.33	213	58.73	0.26	0.0044
22WEd-011	66.33	225	74.63	0.29	0.0040
27WEb-003	76.33	225	114.75	0.34	0.0030
34WEa-004	44.67	289	42.83	0.15	0.0036
EN57AKŚ-223	29.00	203	64.77	0.14	0.0022
EN75-004	No data available				
EN76-009	No data available				

## Data Availability

Not applicable.
